# Inflammation and cognitive performance in elite athletes: A cross-sectional study

**DOI:** 10.1016/j.bbih.2024.100872

**Published:** 2024-10-05

**Authors:** Kati Wiedenbrüg, Laura Will, Lukas Reichert, Sebastian Hacker, Claudia Lenz, Karen Zentgraf, Markus Raab, Karsten Krüger

**Affiliations:** a Martin-Luther University Halle-Wittenberg, Germany; b German Sport University Cologne, Germany; c Goethe University Frankfurt am Main, Germany; d Justus-Liebig University Giessen, Germany

**Keywords:** Executive functions, Processing speed, Inflammation, Metabolic biomarkers, Subjective stress, Elite athletes

## Abstract

Functional cognition is relevant for athletic success and interdependent with physical exercise, yet despite repeatedly demonstrated inflammatory responses to physical training, there are no studies addressing the relationship between cognition and inflammation in athletes. The aim of this study was to investigate the relationship between cognitive performance and selected inflammatory, and further physiological biomarkers in elite athletes. Data from 350 elite athletes regarding cognitive performance (processing speed, selective attention, working memory, cognitive flexibility), systemic inflammatory markers, metabolic hormones, growth factors, tissue damage markers, and micronutrients (e.g., ferritin, 25-OH-vitamin D), as well as physiological, subjective ratings of recovery and stress were analysed by correlative and multiple regression analyses. Results show that across all athletes variance in processing speed, selective attention, and working memory, could be best explained through a combination of metabolic hormones with physiological and psychological indicators of stress, and in cognitive flexibility through vitamin D levels. Only for the subgroup of athletes from closed-skill sports, the ratio TNF-α:IL-10 significantly contributed to explanation of variance in working memory and cognitive flexibility. In general, found correlations point to the importance of inflammatory balance and sufficient long-term nutrient supply for unaffected cognitive performance.

## Highlights

• Inflammatory markers and cognitive performance correlated significantly in closed-, but mostly not in open-skill sports.
• The ratio TNF-α:IL-10 significantly predicted working memory and cognitive flexibility in closed-skill sports.
• Biomarkers for metabolism (fT3, IGFBP-1, leptin) significantly predicted processing speed and selective attention across all athletes.
• Better cognitive flexibility was associated with higher levels of vitamin D.
• Indicators for recovery and stress (creatinine & psychological ratings) explained variance in processing speed and working memory.

## 1 Introduction

Inflammation and cognition – each is a broad research field in its own right, not only because of the complexity of the immune system with its reciprocal and bi-directional interaction to the nervous and the endocrine system (Besedovsky & del Rey, 1996; Rivest, 2010), but also because cognition is multi-faceted, ranging from basic, bottom-up functions like perception or processing speed to higher, top-down functions, like problem-solving (Kalén et al., 2021).

In elite sports, both inflammation and cognition are crucial for performance. The immune system's acute reactions and its long-term adaptations to the constant and intense training usually contribute to recovery and the restoration of homeostasis (Cerqueira et al., 2019; Małkowska and Sawczuk, 2023). Yet, in some cases these inflammatory responses increase susceptibility to infection and injury, which again compromise performance (Posabella, 2020). Cognitively, disciplines differ in their demands and have therefore been subject to different sport classification systems. One of these distinguishes open-from closed-skill sports (Singer, 2000). While open-skill sports require anticipation and rapid reactions to dynamic environments (e.g., basketball, volleyball or ice hockey), closed-skill sports are self-paced and therefore contain the possibility to implement routines before acting (e.g., gymnastics, swimming or horse racing). Even though group differences in cognitive performance were reported in favour of open-skill sports (Koch and Krenn, 2021; Lai et al., 2024), success at elite level was generally positively associated with cognitive functions (Furley et al., 2023; Ishihara et al., 2021; Kalén et al., 2021; Scharfen and Memmert, 2019; Voss et al., 2010). When speaking of cognitive functions, the concept used most widely is that of executive functions (EF), which are considered a set of effortful mental skills enabling concentration, attention and goal-directed behaviour (Diamond, 2013; Friedman and Miyake, 2017). Higher-executive functions such as reasoning, problem-solving, planning and monitoring, are thought to be built upon three core EFs, namely inhibition, working memory (WM) and cognitive flexibility (CF) (Diamond, 2013; Lemke and Scherpiet, 2015). This tripartition of EFs is also discernible neurologically, with EF activating both common and specific neural areas, which can be linked to individual differences in neural activation, volume, and connectivity mainly in the prefrontal cortex and fronto-parietal regions (Friedman and Miyake, 2017; Menon & D'Esposito, 2022; Miller and Cohen, 2001; Schillerstrom et al., 2005). Also, cognition and inflammation are directly and indirectly associated at neural level: Whilst microglia and cytokines have immunomodulatory functions in the brain (Kusnecov and Anisman, 2014; Vitkovic et al., 2000), indirect effects exist through catecholamines, which are relevant for immunomodulation (Gaskill and Khoshbouei, 2022; Thomas Broome et al., 2020) as well as neurotransmission (for an overview see e.g., Logue and Gould, 2014).

Whether such an association between cognition and inflammation is also found at behavioural level has been investigated previously. In general, weak, negative effects of cytokines such as IL-6, IL-1β, IL-10, and TNF-α as well as of acute phase proteins such as CRP on global cognition (Alnefeesi et al., 2020; Bradburn et al., 2017; Fard et al., 2022; Morrens et al., 2022), processing speed and selective attention (Beydoun et al., 2019; Fard et al., 2022; Karagüzel et al., 2019; Mandelblatt et al., 2023; Morrens et al., 2022; Patlola et al., 2023; Tegeler et al., 2016; Tian et al., 2024), and working memory (Alnefeesi et al., 2020; Fard et al., 2022; Morrens et al., 2022; Patlola et al., 2023) have been reported. However, there are also some studies reporting no correlations (Adamowicz et al., 2024; Caldú et al., 2023; Tateishi et al., 2020; Tian et al., 2024; Varanoske et al., 2022), or associations that vanish after adjustment for confounding factors (Slaney et al., 2023; Wennberg et al., 2019).

While those studies demonstrating effects predominantly measured samples with an acute, low-grade, chronic inflammatory status or cognitive decline (i.e., during aging or in the presence of psychiatric disorders), studies measuring healthy participants mostly report conflicting results (Caldú et al., 2023; Slaney et al., 2023). When setting the intersection between cognition and inflammation into the context of exercise, notably no studies for the special population of elite athletes have been published, representing a general research gap. Testing the relationship between EFs and inflammation in such sample, in which inflammation and cognition are central to the profession, enriches and broadens research, which was mainly influenced through studies in clinical, elderly, or samples of average health yet. A (non-)replication of the previous inconsistently-reported relationship in a highly functional sample, moreover, contributes to a further understanding of the interplay of the immune-, the endocrine-, and the neuro system.

In general, confounding variables must be considered when the link between cognition and inflammation is investigated. First, the nutrient supply with especially the micronutrient vitamin D is of relevance. At molecular level, this vitamin not only has immunomodulatory functions but also controls neurotransmitter synthesis (Turkmen et al., 2023; Wang et al., 2023). At behavioural level, vitamin D-levels within normal range have been found to be beneficial for global cognition and EFs in elderly (Alnefeesi et al., 2020; Chen et al., 2023; da Silva et al., 2022; Turkmen et al., 2023), yet not consistently for young and middle-aged participants (e.g., Sahasrabudhe et al., 2020, or review by Redlich et al., 2024).

Second, energy supply on a macronutrient level is relevant: Detrimental effects on EFs and on immunity have been reported for cases of relative energy deficiency in sports (REDs; see e.g., Mountjoy et al., 2023). Also, prolonged and/or severe low energy availability can alter levels of biomarkers for energy balance, such as leptin, free triiodothyronine (fT3), the insulin-like growth factor 1 (IGF-1) and the insulin-like growth factor binding-protein 1 (IGFBP-1; Mountjoy et al., 2023). These again have modulatory effects on inflammation (de Luca et al., 2020; Lasa and Contreras-Jurado, 2022; Procaccini et al., 2012; Witkowska-Sędek and Pyrżak, 2020) and have been associated either with lower global EF performance across clinical samples (Gunstad et al., 2008; Macaluso et al., 2022; Nozari et al., 2023) or with brain development and cognitive function (Alcaide Martin and Mayerl, 2023; Eslami-Amirabadi and Sajjadi, 2021; Frater et al., 2018), respectively. Lastly, it should be mentioned that EFs were found to be negatively associated to the body-mass-index (BMI) (Caldú et al., 2023; Macaluso et al., 2022; Spyridaki et al., 2016). Yet, the BMI is neither a reliable indicator for body composition nor for long-term energy supply in elite athletes (Ode et al., 2007; Torstveit and Sundgot-Borgen, 2012).

Finally, especially in the sports context the state of recovery and stress should be considered as confounder. For both inflammation (Cerqueira et al., 2019; Gleeson and Pyne, 2016; Merle et al., 2022; Nieman et al., 2012; Sellami et al., 2021; Simmons et al., 2021) and EFs (Haverkamp et al., 2020; Huang et al., 2022; Ishihara et al., 2021; Verburgh et al., 2014; Zheng et al., 2021) intensity- and duration-dependent relationships to physiological stress were reported. Corresponding, a relationship between subjective stress and inflammation (see e.g., Di Battista et al., 2020; Souglis et al., 2023) or EFs (see e.g., Plieger and Reuter, 2020) was observed in athletes.

Taking together, we aim at investigating the relationship between selected inflammatory biomarkers and cognitive functions – namely processing speed, selective attention, working memory and cognitive flexibility - in a sample of elite athletes. Moreover, we incorporate other physiological (metabolism, micronutrient supply, stress) and psychological (recovery and stress) markers in our correlative and regressive approach. Based on previous literature, we expected small-to-medium correlational effects between common inflammatory markers and cognitive functions, if any. Moreover, following the gist of actual theories on EF, we await a directional effect from physiological load biomarkers and subjective stress ratings on cognitive performance. Based on the role of energy availability in cognition, inflammation, and exercise, we predict a directional effect of metabolic biomarkers on cognitive performance in our sample of elite athletes. Last, due to lower construct complexity for basic cognition, we generally expect stronger effects for basic than for higher cognitive functions (see Sanbonmatsu et al., 2020 for the role of complexity in psychological research).

## 2 Method

### 2.1 Participants

In our study data from 350 elite athletes (184 female; *M*_age_ = 20.2 ± 4.6 years) were included. All athletes are part of the national squad at junior, intermediate, or Olympic level and perform on international level. Access to athletes was provided by the respective sport association for the disciplines Artistic Gymnastics (*n* = 23), 3x3 Basketball (*n* = 38), Ice Hockey (*n* = 68), Modern Pentathlon (*n* = 32), Trampoline Gymnastics (*n* = 33), Rhythmic Gymnastics (*n* = 28), Table Tennis (*n* = 18), and Volleyball (*n* = 110). The study protocol was approved by the university's local ethics committee (number: AZ 55/22) and was in accordance with the Declaration of Helsinki for human research. Prior to the study, all participants, or their parents (when athletes were underage), received detailed written and verbal elucidation and signed informed consent.

### 2.2 Setup

Data collection took place during training camps or squad meetings and separate seminar rooms were converted into lab space. Demographical information (sex, age, discipline) was provided by the sport associations a priori. On measurement day, athletes first filled in the Short Recovery and Stress Scale for Sport (SRSS; Hitzschke et al., 2015), assessing subjective recovery (physical and mental capability, emotional even temper, general recovery) and subjective stress (muscular stress, lack of activation, emotional uneven temper, general stress) on a 7-point Likert-Scale (0: does not apply at all – 7: applies completely) by one item each. Besides, anthropometric data (height, weight) was collected, and the body-mass-index (BMI) was calculated.

### 2.3 Cognitive tasks

Participants first completed one paper-pencil task in a group session, and then three computerized cognitive tasks individually. For the computer tasks, participants were seated at a table with an approximate viewing distance of 40 cm from a Surface GO 3 tablet wearing noise-cancelling headphones. They were asked to use a tablet pencil to complete the digitalized tasks.

#### 2.3.1 Number-connection task (“Zahlenverbindungstest”, ZVT)

The paper-pencil group task was the Number-Connection Task (ZVT; Oswald, 2016), a German equivalent to the Trail-Making-Test A. This test is validated and is commonly used to measure cognitive processing speed as a basic cognitive function not only in elite sports. Participants were asked to complete two practice trials connecting numbers from 1 to 20 as fast as possible. They were instructed that consecutive numbers always appear in immediate proximity, that the beauty of the strokes was not important and that the only thing that counted was working as fast as they could with the greatest effort. Participants then completed four test pages trying to connect numbers from 0 to 100 in ascending manner as fast as possible. Participants had 30 s to complete each page and the experimenter stopped the time and controlled adherence to the instructions. The dependent variable is the average amount of numbers connected over the four pages, with a higher score representing higher processing speed. Scores were further converted into age-matched standardized values according to norm tables of the manual.

#### 2.3.2 d2-R

Selective attention as one component of the EF inhibition was measured by the electronic version of the d2-R (Brickenkamp et al., 2010), a validated and economic concentration and attention test commonly used in German-speaking research. In this task, participants work through rows of letters randomly composed of d's and p's. Each d or p is surrounded by one or two dashes. Participants must only cross out d's surrounded by two dashes (i.e., target) and ignore irrelevant stimuli (I.e., p's and d's with one dash). Participants completed 5 practice trials and were then instructed to complete 14 screen pages each containing 60 objects. Each screen page automatically ended after 20 s. The dependent variable of interest for this study was the total score, which was calculated by subtracting the number of wrongly marked objects from the number of correctly marked targets. Thus, a high total score represents high selective attention (i.e., good inhibition). The dependent variable was converted into a standardized value according to age-matched norms of the German population (Brickenkamp et al., 2010).

#### 2.3.3 Backward corsi block tapping (BCT)

The Backward Corsi Block Tapping (BCT) operationalized performance in visuo-spatial Working Memory (WM). In each trial, participants saw nine pink squares randomly arranged on a dark background. At the beginning of each trial, individual squares lit up yellow in a certain order. Participants were asked to remember in which order the squares lit up and respond by clicking on the squares in reverse order (i.e., clicking first on the square that lit up last). The number of enlightened squares increased with each two successful rounds of completion. The minimum sequence was two and the maximum sequence was eight squares (i.e., span 2–8). The task ended once participants failed to correctly remember a sequence twice and their total score was then calculated as the product of the longest correctly rehearsed span length times the number of correctly performed trials. The so-called Corsi product score therefore ranged from 0 to 112, with a higher product score representing higher WM capacity.

#### 2.3.4 Cognitive flexibility puzzle (CFP)

The CFP (Gonzalez et al., 2013) is a validated test to measure cognitive flexibility in terms of a person's ability to abandon one cognitive strategy in favour of another based on a change in task demands. The task draws from both the Winsconsin Card Sorting Test and the Trail-Making-Task. Participants had to navigate through a grid of tiles with the tablet pencil. Tiles differed from each other in terms of shape (octopus, fish, or cancer), colour (pink, red, or yellow) and background colour (green, dark blue, or turquoise). At the beginning of each puzzle, the first tile occurred in the upper left corner of the screen, followed by three possible tiles to continue with upon clicking on the first tile. One of the three tiles matched the first tile in shape, shape colour or background colour and enabled participants to succeed with three new tiles. The other two were dead ends and produced an error sound when participants clicked on them. They disappeared when participants proceeded with the new tile. Thus, participants had to identify a match rule with each move and eventually adopt a new rule based on the tiles available. Match rules were valid for a maximum of three tiles. The first puzzle was the familiarization phase. After that, participants completed six puzzles. Each puzzle was randomly generated, with switch moves and order of presentation randomized to mitigate any order effects. The dependent variable was the switch cost, which was calculated by subtracting the average time (in milliseconds) in switch moves from the average time (in milliseconds) in repetition moves. Thus, lower switch costs represent higher cognitive flexibility.

### 2.4 Inflammatory and physiological parameters

All blood samples (peripheral, venous blood; about 25 mL) were drawn by medical staff before noon. The athletes followed their usual diet the days before and on measurement day and came to blood collection in a fed state (breakfasted). Generally, athletes did not train prior to blood sampling on measurement day, leading to a time gap between last training and sampling of at least 10 h.

From EDTA-blood we conducted a complete blood count (CBC; in 10ˆ9/litre) using a cell counter (Sysmex Hematology Analyzer, Norderstedt, Germany). The cytokines Interferon Gamma (IFN-γ), Tumor Necrosis Factor Alpha (TNF-α), the interleukines (IL) IL-1β, IL-6, IL-10, IL-17A as well as Insulin-like Growth Factor Binding Protein 1 (IGFBP-1), Insulin, Leptin/OB, Growth Hormone (GH) and Brain-derived Neurotrophic Factor (BDNF) were analysed with the Luminex® Multiplex Assay Kit using a Luminex (LX)-200 instrument. Creatinine-Kinase (CK; U/l), free Triiodothyronine (FT3; pmol/l), Ferritin (in ng/ml), Vitamin B12 and 25-OH-Vitamin D (both in ng/ml) were analysed by chemiluminescent immunoassay (CLIA). Creatinine (in mg/dl) was analysed by photometric methods and C-Reactive Protein (CRP; in mg/dl) by immunoturbidimetric analysis.

### 2.5 Statistical analysis

For statistical analyses we used IBM SPSS Statistics 29. We checked data for unusual cases using the command DETECTANOMALTY, and rechecked reported cases for plausibility – implausible cases, mainly those with switch costs >1200 ms, were marked and excluded from analyses. For each variable descriptive statistics (mean, standard deviation, median, extreme values, kurtosis) were analysed. We tested metric variables for normal distribution (Shapiro-Wilk-Test) and compared physiological parameters for differences regarding sex and sport type using Mann-Whitney-U-Test. Here, disciplines were subgrouped to closed-skill (Artistic Gymnastics, Modern Pentathlon, Trampoline Gymnastics, Rhythmic Gymnastics) or open-skill sports (3x3 Basketball, Ice Hockey, Table Tennis, Volleyball). We examined the relationship between EFs and inflammation via non-parametrical correlations (Spearman's Rho). Demographic, endocrine and other physiological variables, as well as the self-reported recovery and stress, were also correlated (Spearman's Rho) with each cognitive test score, and with inflammatory biomarkers. Subsequently, multiple regression analyses were conducted three times (overall; closed-skill; open-skill) for each cognitive task (dependent variables) separately to better understand the contribution of physiological and psychological variables to cognitive performance. To approach exploratorily and to minimize suppressor effects, the method of backwards elimination was selected. Relevant physiological markers were entered in the models (block 1), followed by relevant psychological indicators for recovery and stress (block 2) and demographic variables (age, BMI, sex) in block 3. Decisions for relevance and necessity were based on significant correlations (α ≤ .05) to each respective cognitive task in this sample and were made to provide statistical power through data-driven reduction of predictors. Regression analyses were performed with logarithmic transformed variables to normalize the variables' distributions and to adjust for the differing scales and units, and assumptions of linearity, homoscedasticity, independence, and normality of errors were checked. Missing cases were excluded pairwise from analysis. Levels for significance were set to α ≤ .05 (two-sided). Given the small to medium effect sizes in previous literature, effect sizes were defined as small for *r* ≤ .10 and *R*^*2*^ ≤ .02, and as medium for .3 ≤ *r* ≤ .5 and .13 ≤ *R*^2^ ≤ .26, respectively (referring to Cohen, 1988).

## 3 Results

### 3.1 General relationship between cognitive performance and physiological markers

**Fig. 1:**
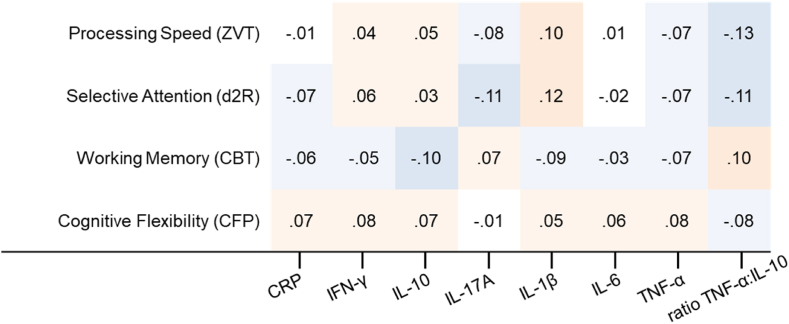
Heatmap for non-parametric correlations (Spearman's Rho) between inflammatory markers and cognitive performance across all athletes. *Note:* Colours indicate the direction (blue = negative, red = positive) and strength (dark = strong; white = none) of the correlations. Significance for α ≤ .05 (two-sided) would be marked by ∗. (For interpretation of the references to colour in this figure legend, the reader is referred to the Web version of this article.)

Results from multiple regression analyses (backwards) calculated across all athletes are provided in Table 1. In general, no inflammatory marker was included in regression analysis due to non-correlation with the dependent variable across all athletes (see Fig. 1). Corrected coefficients of determination (*R*^2^) for higher cognitive functions (selective attention, WM, CF) reached from 1.1% to 9.2%. Most variance (11.1%) was explained for processing speed through a prediction by metabolic markers (IGFBP-1, creatinine, fT3). Physiological indicators for stress and metabolism (creatinine, creatine kinase, urea, IGFBP-1, fT3) were significant predictor variables in the regression models on processing speed, and WM. Standardized beta coefficients indicated worse processing speed and enhanced WM, when metabolic indicators were high. Leptin contributed significantly to explanation of variance only in selective attention. Vitamin D was only included in regression on CF, and beta coefficient indicate better cognitive performance for higher vitamin concentrations. However, the model did not explain substantial variance (*R*^2^ = .011). Subjective, psychological ratings of recovery and stress were included in models predicting worser processing speed (physical capability), lower selective attention (muscular stress), and enhanced WM (emotional uneven temper), but eliminated due to non-significance during backwards regression (processing speed, selective attention).

**Table 1:** Results from multiple regression (backwards elimination) calculated across all athletes for each tested cognitive domain.

Domain	Corrected R^2^	Predictor Variables	Unstandardized Beta (95% CI)	Standardized Beta
Processing Speed	.110,	IGFBP-1	b = - .02 (-.03, - .01)	β = - .241, *p* = .006
	*p =* .002	Creatinine	b = - .11 (-.19, - .03)	β = - .260, *p* = .009
		fT3	b = - .08 (-.15, - .01)	β = - .206, *p* = .018
		Age	b = - .04 (-.12, .03)	β = - .100, *p* = .269
		Sex	b = .00 (-.01, .02)	β = .037, *p* = .705
	.111^a^,	IGFBP-1	b = - .02 (-.03, - .01)	β = - .251, *p* = .004
	*p* = .001	Creatinine	b = - .11 (-.20, - .03)	β = - .268, *p* = .007
		fT3	b = - .08 (-.14, - .01)	β = - .197, *p* = .023
Selective Attention	.092^b^,	Urea	b = - .03 (-.08, .02)	β = - .095, *p* = .258
	*p* = .002	Ferritin	b = - .02 (-.04, .01)	β = - .138, *p* = .124
		Leptin	b = .01 (.00, .03)	β = .157, *p* = .038
		Muscular Stress	b = - .01 (-.04, .01)	β = - .073, *p* = .336
		BMI	b = - .02 (-.15, .10)	β = - .029, *p* = .738
		Age	b = .08 (.01, .14)	β = .174, *p* = .036
		Sex	b = .01 (-.00, 0,26)	β = .163, *p* = .083
	.075^c^,	Leptin	b = .01 (.00, .02)	β = .158, *p* = .036
	*p* < .001	Sex	b = .02 (.01, .03)	β = .259, *p* < .001
Working Memory	.052^d^,	Creatinine	b = .22 (-.01, 1.33)	β = .221, *p* = .054
	*p =* .038	Emotional Uneven Temper	b = .21 (.02, .43)	β = .212, *p* = .032
		Sex	b = .14 (-.05, .19)	β = .136, *p* = .136
	.048^e^,	Creatinine	b = .15 (-.12, 1.02)	β = .151, *p* = .121
	*p* = .029	Emotional Uneven Temper	b = .23 (.04, .45)	β = .233, *p* = .017
Cognitive Flexibility	.011^f^,	Vitamin D	b = - .16 (-.33, .02)	β = - .112, *p* = .075
	*p* = .095	BMI	b = - .26 (-.84, .31)	β = - .056, *p* = .371
	.011^g^, *p* = .048	Vitamin D	b = - .17 (−.24, −.00)	β = - .123, *p* = .048

*Note:* Two models per cognitive task are reported in this table. Model selection followed the criteria of highest R^2^ and/or least number of predictors. 95% Confidence Intervals (CI) are reported for unstandardized coefficients. Reported values refer to log-transformed data. Variables excluded from each model are.

a Physical Capability, BMI, Age, Sex.

b Creatine Kinase, Creatinine.

c Creatine Kinase, Creatinine, Urea, Ferritin, Muscular Stress, BMI, Age.

d fT3, Urea, Physical Capability, General Recovery, BMI, Age.

e fT3, Urea, Physical Capability, General Recovery, BMI, Age, Gender.

f Age, Gender.

g Age, BMI, Gender.

Selected results from exploratory, correlative analyses between cognitive performance and physiological markers across all athletes are presented in Table 2. In general, all *p-*values for the correlations between cognitive performance and inflammatory markers, growth hormone, vitamin B12, and insulin exceeded .05. Regarding anthropometric data, BMI was only associated significantly with performance in WM (*r*(254) = - .180, *p* = .004) across all athletes. Age did not correlate with any cognitive performance, which was expected due to age specific standardization of the tests (see methods).

**Table 2:** Non-parametric correlations (Spearman's Rho (N)) between other physiological markers and cognitive performance across all athletes.

	Processing Speed (ZVT)	Selective Attention (d2R)	Working Memory (CBT)	Cognitive Flexibility (CFP)
IGFBP-1	−.**191**∗ (193)	−.108 (198)	−.006 (154)	.009 (189)
Leptin	.048 (239)	.**134**∗ (244)	−.047 (185)	.007 (234)
fT3	−.071 (323)	**−.153∗** (328)	−.**135**∗ (255)	−.009 (318)
Creatine Kinase	−.115 (270)	−.**175**∗ (275)	−.089 (210)	.002 (265)
Creatinine	−.**261**∗ (269)	−.**176**∗ (273)	−.**239**∗ (208)	−.071 (263)
Urea	−.108 (270)	−.**161**∗ (275)	−.**161**∗ (210)	.011 (265)
Vitamin D	−.100 (270)	−.010 (275)	−.041 (210)	−.**175**∗ (265)
Ferritin	−.049 (270)	−.**163**∗ (275)	.001 (210)	−.018 (265)

*Note*: Significance for α ≤ .05 (two-sided) is marked by ∗.

### 3.2 General relationship between cognitive performance and subjective ratings

Some ratings of subjective recovery and stress correlated with cognitive performance across all athletes. Specifically, processing speed correlated with physical capability (*r*(215) = −.122, *p* = .074). Further, correlations were found between WM and physical capability (*r*(149) = - .220, *p* = .007), general recovery (*r*(149) = - .166, *p* = .043), and emotional uneven temper (*r*(149) = .137, *p* = .096). Selective attention correlated only to muscular stress (*r*(218) = - .150, *p* = .027). No correlations were found between cognitive performance and ratings of mental capability, emotional even temper, lack of activation, and general stress. Correlations between physiological parameters and ratings of recovery and stress are provided in supplementary Table C. In general, subjective ratings correlated with no physiological marker (emotional even temper), only with inflammatory markers (physical capability, mental capability, lack of activation), solely with metabolic hormones or molecules (muscular stress, emotional uneven temper) or with both inflammatory and other physiological parameters (general recovery, general stress). Effect sizes were small.

### 3.3 Subgroup-specifical findings for cognitive performance, physiological markers, subjective ratings and their relationship

Cognitive performance differed significantly between male and female (processing speed, selective attention, WM; *p* < .001). In addition, there were significant differences between male and female participants for CRP, creatine kinase, creatinine, urea, ferritin, vitamin D and insulin (see Supplementary Table A). Sex-specific correlations indicated similar results for ferritin and vitamin D in both sexes. Significant correlations were found only in men for urea (processing speed: *r*(130) = −.217, *p* = .013; selective attention: *r*(132) = −.203, *p* = .020; WM: *r*(108) = −.189, *p* = .049) and creatinine (processing speed: *r*(129) = −.380, *p* < .001; WM: *r*(108) = -. 409, *p* < .001). For CK, all *p*-values exceeded .05 in men and women, when calculated sex-specifically. Regarding the subjective ratings, there were differences between male and female in terms of mental capability, even and uneven emotional temper, and lack of motivation (see supplementary Table A). When correlations were run separately for one sex, mental capability was the only one of these dimensions correlating significantly with cognitive performance (CF) in male (*r*(126) = .184, *p* = .039). All other correlations between these dimensions and cognitive performance were similar (non-significant and not exceeding small effect sizes) for male and female.

Cognitive performance differed significantly between closed-skill and open-skill sports only in regard to selective attention (*p* = .007). Significant differences between these groups were present for the physiological markers creatinine, urea, leptin, vitamin B12 and CRP, but not for any rating of subjective recovery and stress (see Supplementary Table B). When correlations were run separately for one type of sport, only in the group of open-skill sports significant relationships were found for processing speed and IGFBP-1 (*r*(124) = −.295, *p* < .001), creatinine (*r*(202) = −.370, *p* < .001), urea (*r*(203) = −.173, *p* = .014), and vitamin D (*r*(203) = −.146, *p* = .037), between selective attention and ft3 (*r*(226) = −.188, *p* = .004), creatine kinase (*r*(205) = −.177, *p* = .011), creatinine (*r*(203) = −.286, *p* < .001), urea (*r*(205) = −.246, *p* < .001) and ferritin (*r*(205) = −.145, *p* = .038) as well as between CF and vitamin D (*r*(197) = −.178, *p* = .012).

**Fig. 2:**
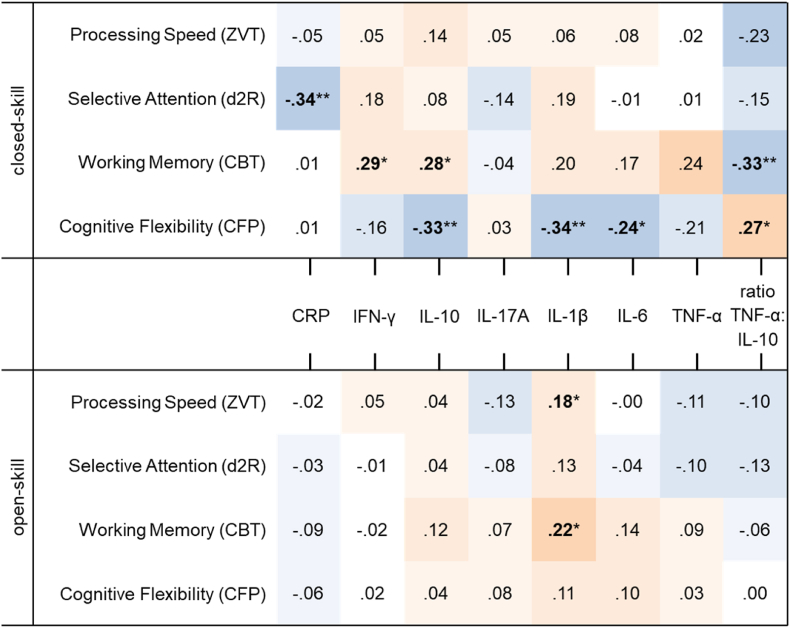
Heatmap for sport-specific, non-parametric correlations (Spearman's Rho) between inflammatory markers and cognitive performance. *Note:* Colours indicate the direction (blue = negative, red = positive) and strength (dark = strong; white = none) of the correlations. Significance for α ≤ .05 (two-sided) is marked by ∗ and for α ≤ .01 (two-sided) by ∗∗. (For interpretation of the references to colour in this figure legend, the reader is referred to the Web version of this article.)

Moreover, significant correlations between cognitive performance and inflammatory markers were found in both groups (see Fig. 2). However, in each linear regression analysis these were excluded during backwards elimination - except in the models predicting WM and CF in the group of closed-skill sports. Here, 14.0% and 5.6% of variance was explained by the ratio TNF-α:IL-10 (WM: β = −.395, *p* = .003; CF: β = .269, *p* = .039). Further, in the group of closed-skill sports processing speed and selective attention were best predicted by sex and BMI or solely by sex. However, neither model explained substantial variance (*R*^2^ = .079, *p* = .024; and *R*^2^ = .072, *p* = .044, respectively). Linear regressions in the group of open-skill sports, showed that variance in processing speed (*R*^2^ = .193, *p* < .001) and CF (*R*^2^ = .015, *p* = .050) was best explained by the physiological markers creatinine (β = −.370, *p* < .001) and IGFBP-1 (β = −.275, *p* = .002) or vitamin D (β = −.141, *p* = .050), respectively. Subjective ratings, particularly emotional even temper (β = .285, *p* = .033), significantly predicted performance in WM in the group of open-skill sports (*R*^2^ = .064, *p* = .033). For processing speed in the group of open-skill sports (*R*^2^ = .074, *p* = .020) all predictors except sex were excluded during backwards elimination.

## 4 Discussion

The aim of this study was to investigate whether a cognition-inflammation-relationship of small to medium effect could also be found in the context of elite sports in regard of possible confounders for cognitive performance. The current study indicates that in a sample of elite athletes, cognitive performance (processing speed; selective attention; working memory, WM) could be best explained through a combination of metabolic hormones with physiological and psychological indicators of stress, and cognitive flexibility (CF) through vitamin D. Results separated for sport type suggest, that these overall results were mainly driven by relationships found in athletes from open-skill sports. In the group of closed-skill sports the ratio TNF-α:IL-10 contributed to explaining variance in WM and CF. In accordance with our hypotheses, models’ effects were small for higher cognitive functions (selective attention, WM, CF), and small-to-medium for basic cognitive functions (processing speed).

Unexpectedly, no single inflammatory marker, but the ratio TNF-α:IL-10 contributed to explanation of variance in cognitive performance. In general, this contrasts previous findings reporting an association between specific inflammatory markers and cognitive performance in clinical and elderly samples (Bradburn et al., 2017; Fard et al., 2022; Karagüzel et al., 2019; Morrens et al., 2022; Tegeler et al., 2016). Yet, this is in line with studies with healthy participants and soldiers, where no associations between single inflammatory markers and vigilance, EFs or processing speed were reported either (Balter et al., 2019; Piantella et al., 2022; van den Heuvel et al., 2022; Varanoske et al., 2022; Wennberg et al., 2019). Notably, in the study from Piantella et al. (2022) no single inflammatory marker, but the ratio TNF-α:IL-10 was negatively associated with attention and WM in jockeys. Considering that this ratio is an indicator for inflammatory imbalance, the results by Piantella et al. (2022) as well as our study suggest that it is this imbalance, and not the pro- or anti-inflammatory processes per se, that affect cognition. Restrictively, this explanatory hypothesis just seems to apply to closed-skill sports, but further studies for verification or falsification are missing so far. It therefore can only be speculated whether in the group of open-skill sports other, unmeasured confounders (e.g. non-/training-related stress, energy availability) levelled out the correlation, or whether in open-skill sports the inflammatory imbalance actually is not associated to cognitive performance.

Especially for the group of open-skill sports, results confirmed the importance of nutrient and energy supply for basic and higher cognition: On micronutrient level, higher levels of vitamin D were associated with lower switch costs in the cognitive flexibility task, which supports the previously reported beneficial effects on executive functions (Chen et al., 2023; da Silva et al., 2022; Turkmen et al., 2023). However, as vitamin D did not correlate with inflammatory markers in our sample, a mediation through the vitamins immunomodulatory functions is less likely than an influence on cognition through affecting neurological mechanisms, like neurotransmitter synthesis (Wang et al., 2023). Notably, two thirds of our sample had insufficient or deficient vitamin D levels. Considering this, the positive effect of vitamin D on CF is consistent with threshold-like approaches, which state that vitamin D is beneficial in cases of insufficiency and deficiency, but that supra-threshold levels do not contribute further to cognitive performance (Chen et al., 2023). However, it is inconclusive, why we only found correlations of vitamin D and CF but not to other cognitive functions, because higher cognitive functions should have similar neurological activations (see e.g., Barbey et al., 2013). Therefore, we suggest not to overestimate the correlation of vitamin D to CF in this sample. As there are not many studies about the effect on cognition especially in younger and middle-aged, and those existing are inconclusive (Redlich et al., 2024), the role of vitamin D in cognition should be investigated in further studies. In the light of our results, implementing tests with higher sensitivity and specificity to higher cognitive functions is advisable.

On macronutrient level, hormones and molecules relevant in energy metabolism, like insulin-like growth factor binding protein 1 (IGFBP-1), and creatinine, respectively, influenced cognitive performance, especially where fast and correct work is of major role. Higher levels of IGFBP1 predicted slower processing speed in elite athletes from open-skill sports and across all athletes. It is known that insulin-like growth factor 1 (IGF-1) and its binding protein are relevant for metabolism, growth and neurogenesis (Bach, 2018; Frater et al., 2018). At behavioural level, higher IGF1-levels were related with better executive functioning (elderly: Al-Delaimy et al., 2009; Bellar et al., 2011; Frater et al., 2018), and higher IGFPB-1 levels with slower processing speed (patients with multiple sclerosis: Butti et al., 2022). One explanation for this association was provided by Butti et al. (2022), who pointed to the subventricular zone, which not only secretes IGFBP-1, but influences striatal functioning and processing speed. Although our study replicated findings about the association of IGFBP-1 and processing speed in a young and healthy sample of elite athletes, it did not verify neurological explanations. Therefore, it might be fruitful to have a closer look at IGFBP-1 in the context of processing speed in further research.

A higher concentration of creatinine was related to lower processing speed in elite athletes and the subgroup of open-skill sports. Its precursor is creatine, which provides energy not only to muscle cells but also to the brain. In previous studies the importance of creatine mainly for reaction time and short-term memory has been showed (Avgerinos et al., 2018; Pires et al., 2021; Roschel et al., 2021). Suggested explanations for the beneficial effects of creatine on cognition are neurotransmission (Meftahi et al., 2023), neuroprotection and energy provision (Avgerinos et al., 2018). Coming to creatinine, however, there are no studies investigating the role of creatinine for cognition yet. It is speculated that the reduction of creatine and its beneficial, energy-supplying effects, or that creatinine itself, have restricting effects on the brain, which would explain its diminishing effects on cognitive functions in our study. Especially in the context of sports, where creatine supplementation trends (see e.g., Forbes et al., 2023 or Hall et al., 2021), we recommend to incorporate creatinine in future research and to regard possible diminishing effects of creatines metabolite, creatinine.

On a subjective level, ratings for recovery and stress contributed to explanation of variance in processing speed and working memory in elite athletes. Having a closer look, it was not mental, but physical/muscular and general recovery as well as emotional even temper contributing to cognitive performance in all athletes and in athletes from open-skill sports. This result is noteworthy, as previous studies mostly investigated either the influence of (experimentally-induced) mental fatigue on cognitive or physical performance (Dong et al., 2022; Habay et al., 2021; Holgado et al., 2020) or of physical fatigue on physical performance (Dambroz et al., 2022; Pageaux and Lepers, 2016), but rarely the influence of subjective, physical, or general recovery on cognitive performance. In contrast to experiments about acute exercise bouts on EFs (e.g., Huang et al., 2022; Ishihara et al., 2021; Zheng et al., 2021; Zhu et al., 2021), we did not examine cognitive performance following an acute exercise bout but had insight to the athletes’ current perceptions in midst of their training camps or everyday sporting life through the questionnaire. Even though there are similar findings about subjective, overall stress and recovery ratings in other contexts (Fan and Smith, 2017; Ye and Pan, 2016), our results are rather novel in the context of sports. Integrating subjective ratings is a major strength of this study and enriches research to cognitive functioning. Therefore, collecting subjective ratings in further research to mental and physical stress especially in the sports context is advisable, considering that subjective ratings are economic to collect, are an important completion to physiological markers, and, in this study, contributed to explanation of variance in processing speed and working memory.

Overall, this study illustrates the relevance of nutrient and energy supply through objective physiological and subjective psychological markers for cognitive performance in general, but especially for basic cognitive processes. Unexpectedly, inflammation had a negligible effect on cognitive performance across all athletes. As this was in line with previous research in young and healthy samples and in contrast to results from clinical samples, results point to threshold-like explanatory approaches and militate for the importance of allostasis for unaffected cognitive performance.

### 4.1 Limitations and strengths

Comparing these results to a sedentary group might have been interesting, indeed studies investigating inflammation and cognitive performance have already been conducted in other areas (see e.g., Adamowicz et al., 2024; Balter et al., 2019; Beydoun et al., 2019; Caldú et al., 2023; Lin et al., 2018; Slaney et al., 2023; Varanoske et al., 2022). Furthermore, this relationship could be highly special in elite athletes, as a high amount of physical activity and mental workload is characteristic for this group. In addition, our study suggests that even within the group of elite athletes, the inflammation-cognition-relationship is different between open- and closed skill sports.

A limitation is that we did not collect physiological indicators for stress, like glucocorticoids or catecholamines. We decided against collecting these indicators, because in cross-sectional studies like ours, a single measurement of physiological stress indicators is strongly influenced by other factors, like time of day, and interpretation of results would have been limited (Luecken and Gallo, 2007; Peaston and Weinkove, 2004). Nevertheless, consideration of stress-related physiological markers might have fulfilled the picture about the inflammation-cognition-relationship, through their connection to both cognition (see e.g., meta-analysis by Shields et al., 2016) and inflammation (see e.g., Rohleder, 2019). Besides, if we had measured cortisol, this would have enabled us to identify deviant cases on this level, such as cases of relative energy deficiency in sports (REDs).

Also, it was not optimal to operationalise inhibition via the d2R-test for concentration and (selective) attention, which is a popular and validated measure in German-speaking research and practice. Its popularity led to high test acceptance among athletes, but facilitating effects due to its familiarity cannot be excluded (Daseking and Putz, 2015). Beyond that, the d2-R defines selective attention as fast and correct selection of targets, which overlaps with definitions of processing speed and might have harmed discriminatory power. However, in weighing the disadvantages of the d2R with its advantages, and comparing it to optional cognitive measures for inhibition, like the Stroop-Paradigm, we chose the d2R-test because of its time-efficient test execution and evaluation, and its validation in German and European age-matched samples. Aside from these limitations, our study excels through the characteristics of the sample and the interdisciplinary study design. Characteristics of our sample were participants' elite status (part of the national squad and competing at international level), the comparatively large sample size (*N* = 350), and the young age (20.2 ± 4.6 years). Comparing these sample characteristics to other studies investigating cognition (see e.g., Huang et al., 2022; Scharfen and Memmert, 2019) or inflammation (see e.g., Cerqueira et al., 2019; Gleeson and Pyne, 2016; Simmons et al., 2021) in elite sports context, it is evident that the sample size of the current study is reliable. The current study closes the research gap as the study's participants are best trained and much younger than samples in studies investigating the cognition-inflammation-relationship (see e.g., Bradburn et al., 2017; Fard et al., 2022; Mandelblatt et al., 2023; Morrens et al., 2022).

## 5 Conclusion

Across all elite athletes, the level of inflammatory markers was not associated with cognitive performance, but sufficient nutrient – and energy supply seemed to play a major role especially for basic cognitive functions. Specifically, fT3, leptin, IGFBP-1, creatinine and vitamin D were associated with processing speed, selective attention, and cognitive flexibility, respectively. Analyses specifically for the subgroup of closed-skill sports yielded that the ratio TNF-α:IL-10 significantly contributed to explanation of variance in WM and CF. In addition to those physiological markers, psychological ratings about recovery and stress contributed to the explanation of variance and completed the picture with a subjective dimension, which has received rather less attention in correlational, cross-sectional studies in sports before. This study adds value to research not only by contextualizing those subjective ratings to cognitive performance in athletes, but by demonstrating that the (non-)existing correlations speak for a limited generalizability of the inflammation-cognition-relationship from clinical/elderly to the sample of well-trained elite athletes yet are partly in line with the conflicting results found in healthy participants. Overall, these study's results point to threshold-like explanations and the importance of inflammatory balance as well as sufficient nutrient supply for unaffected cognitive performance.

## Funding source declaration

This research was funded by the National Federal Institute for Sports Science in 2021–2025 (Grant No. 081901/21-25, English title: Individual performance development in elite sports by holistic and transdisciplinary process optimization). The data analysed for the purposes of this study was part of a multidisciplinary large-scale data set, which included multiple points of measurement and a cross-sectional and longitudinal perspective. The subset of data included in this study covered the cross-sectional data collected in the period February 2022 - August 2023.

## CRediT authorship contribution statement

**Kati Wiedenbrüg:** Writing – original draft, Visualization, Project administration, Investigation, Formal analysis, Data curation, Conceptualization. **Laura Will:** Writing – review & editing, Writing – original draft, Supervision, Data curation, Conceptualization. **Lukas Reichert:** Writing – review & editing, Data curation. **Sebastian Hacker:** Writing – review & editing, Data curation. **Claudia Lenz:** Writing – review & editing, Data curation. **Karen Zentgraf:** Supervision, Project administration, Funding acquisition. **Markus Raab:** Writing – review & editing, Supervision, Methodology, Conceptualization. **Karsten Krüger:** Writing – review & editing, Supervision, Project administration, Methodology, Investigation, Funding acquisition, Conceptualization.

## Declaration of competing interest

The authors declare the following financial interests/personal relationships which may be considered as potential competing interests:

The in:prove project (PI: Krüger, K.; Zentgraf, K.) reports financial support was provided by National Federal Institute for Sports Science Science [grant number 081901/21-25]. Ifthere are other authors, they declare that they have no known competing financial interests or personal relationships that could have appeared to influence the work reported in this paper.

## Data Availability

Data will be made available on request.
