# Supplementary material for: Inflammation and cognitive performance in elite athletes: A cross-sectional study
Source: Brain Behav Immun Health. 2024 Oct 5;42:100872. doi: 10.1016/j.bbih.2024.100872 (PMC11776080; doi:10.1016/j.bbih.2024.100872)
Supplement: Multimedia component 1 [file mmc1.docx]

| **Supplementary Table A**  *Descriptive Statistics for cognitive, physiological, psychological, and anthropometric variables for male and female athletes plus levels of significance for group comparisons.* | | | | |  |  |  |
| --- | --- | --- | --- | --- | --- | --- | --- |
|  | Male | Female | | Significance | |  |  |
| **Cognitive Performance^1^** | | |  | |  | |  |
| Processing Speed  Selective Attention  Working Memory  Cognitive Flexibility | 106.08 ± 0.75 [104.60,107.57], *n* = 160  101.86 ± 0.70 [100.47,103.25], *n* = 162  29.92 ± 2.05 [25.87,33.97], *n* = 133  455.10 ± 23.45 [408.78,501.43], *n* = 155 | 109.78 ± 0.71 [108.38,111.17], *n* = 179  106.27 ± 0.64 [105.00, 107.53], *n* = 182  40.98 ± 1.77 [37.48, 44.48], *n* = 138  461.06 ± 23.07 [415.53,506.59], *n* = 177 | | *p* < .001  *p* < .001  *p* < .001  *p* = .808 | |  |  |
| **Inflammatory and Physiological Parameters** | | |  | |  | |  |
| IFN-γ  TNF-α  IL-1β  IL-6  IL-10 | 350.22 ± 83.41 [185.09,515.35], *n* = 122  7.22 ± 0.76 [5.70,8,73], *n* = 98  15.46 ± 1.99 [11.51,19.42], *n* = 123  3.49 ± 0.49 [2.51,4.49], *n* = 98  3.62 ± 0.42 [2.78,4.47], *n* = 98 | 643.39 ± 264.93 [119.07,1167.71], *n* = 126  6.10 ± 0.53 [5.04, 7.16], *n* = 108  15.06 ± 1.57 [11.94,18.18], *n* = 127  2.75 ± 0.37 [2.01,3.49], *n* = 108  3.09 ± 0.24 [2.53,3.65], *n* = 108 | | *p* = .862  *p* = .222  *p* = .749  *p* = .051  *p* = .769 | |  |  |
| **Supplementary Table A. Continued.** | | |  | |  | |  |
|  | Male | Female | | Significance | |  |  |
| **Inflammatory and Physiological Parameters** | | |  | |  | |  |
| IL-17A  Ratio TNF-α:IL-10  IGFBP-1  Insulin  Leptin  Growth Hormone  BDNF  CRP  Creatine Kinase  Creatinine  Urea  Ferritin | 44.49 ± 24.98 [0.00,93.99], *n* = 116  2.84 ± 0.25 [2.34,3.34], *n* = 98  15373.62 ± 1927.69 [11547.18, 19200.05], *n* = 97  503.35 ± 95.09 [314.61,692.08], *n* = 98  2236.79 ± 241.78 [1758.17,2715.41], *n* = 123  4315.89 ± 992.92 [2345.22,6286.56], *n* = 98  1436.20 ± 514.48 [411.07,2461.33], *n* = 75  0.14 ± 0.29 [0.08,0.20], *n* = 130  544.80 ± 42.78 [460.19,629.41], *n* = 134  1.00 ± 0.02 [0.97,1.04], *n* = 133  37.37 ± 0.83 [35.72,39.02], *n* = 134  66.85 ± 3.25 [60.43,73.28], *n* = 134 | 3.89 ± 4.04 [5.89,21.89], *n* = 113  13.46 ± 0.90 [1.67,5.26], *n* = 108  14781.73 ± 1627.70 [11554.29,18009.16], *n* = 106  543.06 ± 47.88 [448.14,637.98], *n* = 108  1931.32 ± 211.83 [1512.12, 2350.53], *n* = 127  3915.82 ± 653.39 [2620.55,5211.10], *n* = 108  544.05 ± 68.52 [407.51,680.59], *n* = 75  0.56 ± 0.01 [0.04, 0.07], *n* = 142  255.25 ± 17.37 [220.91,289.59], *n* = 145  0.80 ± 0.01 [0.78, 0.82], *n* = 144  29.19 ± 0.59 [28.03,30.36], *n* = 145  35.59 ± 2.04 [31.56,39.63], *n* = 145 | | *p* = .461  *p* = .703  *p* = .468  *p* = .049  *p* = .308  *p* = .909  *p* = .891  *p* < .001  *p* < .001  *p* < .001  *p* < .001  *p* < .001 | |  |  |
| **Supplementary Table A. Continued.** | | |  | |  | |  |
|  | Male | Female | | Significance | |  |  |
| **Inflammatory and Physiological Parameters** | | |  | |  | |  |
| Vitamin B12  Vitamin D  fT3 | 471.39 ± 11.78 [448.08,494.70], *n* = 134  28.26 ± 0.98 [26.31,30.19], *n* = 134  5.23 ± 0.06 [5.12,5.35], *n* = 164 | 443.09 ± 18.67 [406.16,480.03], *n* = 145  27.31 ± 0.90 [25.53,29.09], *n* = 145  4.99 ± 0.08 [4.83,5.15], *n* = 170 | | *p* = .041  *p* = .398  *p* = .125 | |  |  |
| **Subjective Ratings (SRSS)^2^** | | |  | |  | |  |
| Physical Capability  Mental Capability  Emotional Even Temper  General Recovery  Muscular Stress  Lack of Activation  Emotional Uneven Temper  General Stress | 3.99 ± 0.10 [3.79,4.19], *n* = 131  4.46 ± 0.08 [4.29,4.63], *n* = 131  4.68 ± 0.10 [4.47,4.87], *n* = 131  3.63 ± 0.09 [3.45,3.81], *n* = 131  2.58 ± 0.12 [2.35,2.81], *n* = 131  1.24 ± 0.11 [0.92,1.35], *n* = 131  1.39 ± 0.11 [1.17,1.63], *n* = 131  2.25 ± 0.12 [2.01,2.48], *n* = 123 | 3.92 ± 0.09 [3.72,4.11], *n* = 119  4.18 ± 0.11 [3.97,4.39], *n* = 119  4.28 ± 0.12 [4.04,4.51], *n* = 119  3.67 ± 0.11 [3.44,3.90], *n* = 119  2.34 ± 0.13 [2.08,2.59], *n* = 119  1.46 ± 0.12 [1.23,1.69], *n* = 119  1.87 ± 0.14 [1.58,2.15], *n* = 119  2.05 ± 0.14 [1.78,2.32], *n* = 99 | | *p* = .244  *p* = .041  *p* = .012  *p* = .674  *p* = .177  *p* = .037  *p* = .036  *p* = .277 | |  |  |
| **Supplementary Table A. Continued.** | | |  | |  | |  |
|  | Male | Female | | Significance | |  |  |
| **Anthropometric Data** |  |  | |  | |  |  |
| BMI | 23.47± 0.19 [23.09, 23.86], *n* = 153 | 21.40 ± 0.18 [21.03, 21.76], *n* = 178 | | *p* < .001 | |  |  |
| *Note:* ^1^Unities are the Average (Processing Speed) and Total (Selective Attention, Working Memory) Amount of Correctly Marked Items, and milliseconds for Cognitive Flexibility (see Methods).  ^2^Dimensions were rated on a seven-point Likert-Scale (0: does not apply at all – 7: applies completely) per one item each. | | | | |  |  |  |

| **Supplementary Table B**  *Descriptive Statistics for cognitive, physiological, psychological, and anthropometric variables for athletes from closed-skill and open-skill sport plus levels of significance for group comparisons.* | | | |
| --- | --- | --- | --- |
|  | Closed-skill | Open-skill | Significance |
| **Cognitive Performance^1^** | | |  |
| Processing Speed  Selective Attention  Working Memory  Cognitive Flexibility | 107.43 ± 8.80 [105.78, 109.09], *n* = 111  102.37 ± 8.56 [100.78, 103.96], *n* = 114  37.94 ± 22.29 [33.65, 42.24], *n* = 106  418.75 ± 402.42 [343.40, 494.09], *n* = 112 | 108.33 ± 10.02 [107.02, 109.64], *n* = 228  105.10 ± 9.19 [103.91, 106.29], *n* = 230  34.02 ± 23.16 [30.46, 37.58], *n* = 165  478.41 ± 228.84 [448.00, 508.82], *n* = 220 | *p* = .424  *p* = .007  *p* = .213  *p* = .747 |
| **Inflammatory and Physiological Parameters** | | |  |
| IFN-γ  TNF-α  IL-1β  IL-6  IL-10 | 840.66 ± 3444.79 [127.26, 1554.05], *n* = 92  6.67 ± 6.79 [5.10, 8.24], *n* = 74  17.01 ± 20.35 [12.80, 21.23], *n* = 92  3.05 ± 4.30 [2.06, 4.05], *n* = 74  3.28 ± 2.92 [2.60, 3.95], *n* = 74 | 297.78 ± 867.15 [160.63, 434.92], *n* = 156  6.61 ± 6.50 [5.49, 7.73], *n* = 132  14.24 ± 19.79 [11.13, 17.35], *n* = 158  3.14 ± 4.50 [2.36, 3.91], *n* = 132  3.38 ± 3.94 [2.70, 4.06], *n* = 132 | *p* = .244  *p* = .910  *p* = .654  *p* = .497  *p* = .407 |
| **Supplementary Table B. Continued.** | | |  |
|  | Closed-skill | Open-skill | Significance |
| **Inflammatory and Physiological Parameters** | | |  |
| IL-17A  Ratio TNF-α:IL-10  IGFBP-1  Insulin  Leptin  Growth Hormone  BDNF  CRP  Creatine Kinase  Creatinine  Urea  Ferritin | 11.56 ± 18.75 [7.49, 15.63], *n* = 84  2.49 ± 1.42 [2.16, 2.82], *n* = 74  15232.87 ± 15887.90 [11551.94, 18913.80], *n* = 74  507.71 ± 486.50 [395.00, 620.42], *n* = 74  1538.97 ± 1994.12 [1126.00, 1951.93], *n* = 92  3335.95 ± 4796.10 [2224.78, 4447.12], *n* = 74  485.50 ± 514.64 [352.56, 618.45], *n* = 60  0.04 ± 0.07 [0.03, 0.06], *n* = 72  365.34 ± 411.24 [268.71, 461.98], *n* = 72  0.82 ± 0.14 [0.79, 0.86], *n* = 72  30.48 ± 7.46 [28.72, 32.23], *n* = 72  48.66 ± 40.60 [39.12, 58.20], *n* = 72 | 39.73 ± 243.20 [-0.19, 79.65], *n* = 145  3.55 ± 8.68 [2.05, 5.04], *n* = 132  14968.00 ± 18890.86 [11676.98, 18259.01], *n* = 129  533.39 ± 852.61 [386.59, 680.20], *n* = 132  2397.58 ± 2760.44 [1963.82, 2831.35], *n* = 158  4537.93 ± 9794.02 [2851.56, 6224.30], *n* = 132  1326.54 ± 4083.23 [471.33, 2181.76], *n* = 90  0.12 ± 0.29 [0.08, 0.16], *n* = 200  404.40 ± 398.20 [349.83, 458.97], *n* = 207  0.93 ± 0.19 [0.90, 0.95], *n* = 205  34.04 ± 9.76 [32.70, 35.38], *n* = 207  51.28 ± 33.11 [46.75, 55.82], *n* = 207 | *p* = .253  *p* = .453  *p* = .787  *p* = .931  *p* < .001  *p* = .847  *p* = .468  *p* < .001  *p* = .459  *p* < .001  *p* = .009  *p* = .075 |
| **Supplementary Table B. Continued.** | | |  |
|  | Closed-skill | Open-skill | Significance |
| **Inflammatory and Physiological Parameters** | | |  |
| Vitamin B12  Vitamin D  fT3 | 492.40 ± 154.14 [453.58, 531.22], *n* = 63  27.36 ± 10.89 [24.80, 29.92], *n* = 72  5.14 ± 1.10 [4.93, 5.35], *n* = 104 | 446.41 ± 188.99 [420.51, 472.31], *n* = 207  27.91 ± 11.18 [26.38, 29.44], *n* = 207  5.10 ± 0.83 [4.99, 5.21], *n* = 230 | *p* = .012  *p* = .980  *p* = .208 |
| **Subjective Ratings (SRSS)^2^** | | |  |
| Physical Capability  Mental Capability  Emotional Even Temper  General Recovery  Muscular Stress  Lack of Activation  Emotional Uneven Temper  General Stress | 3.97 ±1.01 [3.77, 4.17], *n* = 103  4.30 ±1.08 [4.09, 4.51], *n* = 103  4.33±1.35 [4.07, 4.59], *n* = 103  3.63 ±1.18 [3.40, 3.86], *n* = 103  2.41 ±1.36 [2.14, 2.67], *n* = 103  1.42 ±1.30 [1.16, 1.67], *n* = 103  1.73 ±1.55 [1.43, 2.03], *n* = 103  2.10 ±1.37 [1.79, 2.42], *n* = 77 | 3.95 ±1.20 [3.75, 4.14], *n* = 147  4.35 ±1.09 [4.17, 4.52], *n* = 147  4.59 ±1.12 [4.41, 4.77], *n* = 147  3.66 ±1.15 [3.48, 3.85], *n* = 147  2.50 ±1.36 [2.28, 2.73], *n* = 147  1.20 ±1.22 [1.00, 1.40], *n* = 147  1.55 ±1.38 [1.32, 1.77], *n* = 147  2.19 ±1.34 [1.97, 2.41], *n* = 145 | *p* = .610  *p* = .527  *p* = .196  *p* = .630  *p* = .622  *p* = .178  *p* = .478  *p* = .533 |
| **Supplementary Table B. Continued.** | | |  |
|  | Closed-skill | Open-skill | Significance |
| **Anthropometric Data** |  |  |  |
| BMI  Age | 20.79 ± 2.17 [20.37, 21.21], *n* = 106  19.12 ± 4.07 [18.37, 19.88], *n* = 113 | 23.10 ± 2.54 [22.77, 23.43], *n* = 225  20.69 ± 4.80 [20.07, 21.32], *n* = 229 | *p* < .001  *p* < .001 |
| *Note:* ^1^Unities are the Average (Processing Speed) and Total (Selective Attention, Working Memory) Amount of Correctly Marked Items, and milliseconds for Cognitive Flexibility (see Methods).  ^2^Dimensions were rated on a seven-point Likert-Scale (0: does not apply at all – 7: applies completely) per one item each. | | | |

| **Supplementary Table C**  *Non-parametric correlations across all athletes (Spearman‘s Rho (N)) between the dimensions of SRSS with physiological markers.* | | | | | | | | |
| --- | --- | --- | --- | --- | --- | --- | --- | --- |
|  | Physical Capability | Mental Capability | Even Emotional Temper | General Recovery | Muscular Stress | Lack of Activation | Uneven Emotional Temper | General Stress |
| IFN-γ | -.071 (180) | -.134 (180) | -.016 (180) | .070 (180) | -.043 (180) | -.047 (180) | .002 (180) | .028 (170) |
| IL-10 | -.088 (146) | -.133 (146) | -.002 (146) | .159 (146) | -.052 (146) | -.015 (146) | .049 (146) | .082 (142) |
| IL-17A | -.042 (171) | -.109 (171) | -.051 (171) | .034 (171) | -.023 (171) | -.031 (171) | -.019 (171) | .069 (161) |
| IL-1β | **-.160* (181)** | **-.183* (181)** | -.061 (181) | .045 (181) | -.040 (181) | .044 (181) | .103 (181) | .047 (171) |
| IL-6 | -.027 (146) | -.055 (146) | .026 (146) | .150 (146) | -.032 (146) | -.085 (146) | -.103 (146) | .004 (142) |
| TNF-α | .056 (146) | .122 (146) | .047 (146) | **.172* (146)** | -.040 (146) | -.160 (146) | -.111 (146) | -.058 (142) |
| BDNF | .120 (135) | .011 (135) | -.107 (135) | -.021 (135) | .016 (135) | .024 (135) | .063 (135) | .115 (126) |
| Ferritin | -.054 (201) | .010 (201) | .076 (201) | .020 (201) | .070 (201) | -.011 (201) | -.067 (201) | .062 (197) |
| Creatine Kinase | -.016 (201) | .016 (201) | .021 (201) | -.135 (201) | .061 (201) | .065 (201) | -.117 (201) | .071 (197) |
| Creatinine | .078 (200) | .002 (200) | .091 (200) | -.053 (200) | .118 (200) | -.084 (200) | -.096 (200) | **.147* (196)** |
| **Supplementary Table C. Continued.** | | | | | | | | |
|  | Physical Capability | Mental Capability | Even Emotional Temper | General Recovery | Muscular Stress | Lack of Activation | Uneven Emotional Temper | General Stress |
| Urea | .029 (201) | -.030 (201) | .095 (201) | .025 (201) | .127 (201) | -.014 (201) | .010 (201) | **.194* (197)** |
| CRP | .093 (198) | .047 (198) | -.005 (198) | -.058 (198) | .044 (198) | -.001 (198) | .050 (198) | .086 (194) |
| Vitamin B12 | .068 (192) | .068 (192) | .104 (192) | .016 (192) | .014 (192) | -.091 (192) | -.064 (192) | -.077 (188) |
| Vitamin D | -.025 (201) | -.125 (201) | .054 (201) | .010 (201) | **.167* (201)** | .052 (201) | -.031 (201) | .096 (197) |
| fT3 | -.025 (238) | -.058 (238) | .079 (238) | -.005 (238) | -.042 (238) | -.083 (238) | -.086 (238) | .042 (222) |
| Growth Hormone | .053 (146) | .086 (146) | .106 (146) | .138 (146) | -.106 (146) | -.117 (146) | **-.214* (146)** | -.063 (142) |
| IGFBP-1 | .126 (144) | **.177* (144)** | .059 (144) | .126 (144) | -.081 (144) | -.047 (144) | -.163 (144) | -.124 (140) |
| Insulin | -.091 (146) | -.127 (146) | -.048 (146) | .095 (146) | -.080 (146) | -.026 (146) | .124 (146) | -.024 (142) |
| Leptin | -.069 (181) | -.073 (181) | -.021 (181) | .013 (181) | -.008 (181) | -.032 (181) | .065 (181) | -.028 (171) |
| Ratio TNFα: IL-10 | .158 (146) | **.204* (146)** | .011 (146) | -.029 (146) | -.026 (146) | -.080 (146) | **-.169* (146)** | **-.204* (142)** |
| *Note*: Significance for α ≤ .05 (two-sided) is marked by *. | | | | | | | | |
